# Root stem cell niche networks: it’s complexed! Insights from Arabidopsis

**DOI:** 10.1093/jxb/erab272

**Published:** 2021-06-26

**Authors:** Renan Pardal, Renze Heidstra

**Affiliations:** 1 Wageningen University & Research, Plant Sciences department, Plant Developmental Biology group, Droevendaalsesteeg 1, 6708PB Wageningen, Netherlands; 2 University of Antwerp, Belgium

**Keywords:** Arabidopsis, development, JACKDAW, PLETHORA, quiescent centre, root, SCARECROW, SHORT-ROOT, stem cell niche, WOX5

## Abstract

The presence of two meristematic cell populations in the root and shoot apex allows plants to grow indefinitely. Due to its simple and predictable tissue organization, the Arabidopsis root apical meristem remains an ideal model to study mechanisms such as stem cell specification, asymmetric cell division, and differentiation in plants. The root stem cell niche consists of a quiescent organizing centre surrounded by mitotically active stem cells, which originate all root tissues. The transcription factors PLETHORA, SCARECROW, and WOX5 form signalling hubs that integrate multiple inputs from an increasing number of proteins implicated in the regulation of stem cell niche function. Recently, locally produced auxin was added to the list of important mobile factors in the stem cell niche. In addition, protein–protein interaction data elegantly demonstrate how parallel pathways can meet in a common objective. Here we discuss how multiple networks converge to specify and maintain the root stem cell niche.

## Teaser

An updated overview of the main gene networks converging for the specification and maintenance of the Arabidopsis root stem cell niche.

## Introduction

**Fig. 1.**
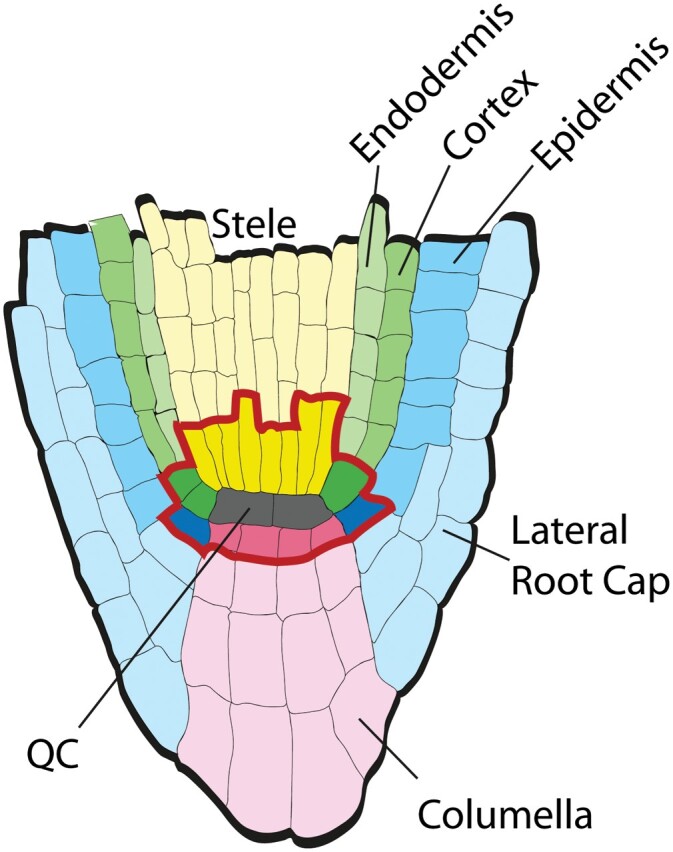
Schematic representation of the Arabidopsis root tip. The stem cell niche, enclosed by the red line, consists of a centrally localized quiescent centre (QC) surrounded by the tissue initials/stem cells. Vascular initials are above the QC, shown in dark yellow. Flanking the QC are two types of initials: the ground tissue initials, shown in dark green that originate the cortex and endodermis, and the epidermis/lateral root cap initials, shown in dark blue. Below the QC, shown in dark pink, are the columella initials.

Unlike animals, plants display indefinite growth and produce organs throughout their life cycle. This is possible due the presence of two populations of stem cells located in the shoot and root apex. The Arabidopsis root apical meristem remains one of the best studied model systems in plants to address developmental questions like stem cell specification, differentiation, regeneration, cell–cell interaction, and cell cycle control. One aspect of the continued popularity of the Arabidopsis root meristem is the highly structured, simple, and predictable tissue organization. The root stem cell niche resides within the meristem and is composed of a group of infrequently dividing cells named the quiescent centre (QC), surrounded by mitotically active stem cells, also called initials (Dolan *et al.*, 1993). Division of the initials produces daughter cells that remain in ordered cell files and together form all the tissues that compose the root in a stereotypic pattern. The initial cells together with the QC are considered a stem cell niche (SCN). Within the SCN, distal to the QC, the columella initials (or distal meristem) divide to form the mature differentiated columella cells. The latter are easily distinguished from the columella initials by the accumulation of starch-laden amyloplasts. Proximal to the QC are the vascular initials and the ground tissue initials. The vascular initials originate the stele after going through a round of formative division. The ground tissue initial daughter originates both cortex and endodermis. Finally, lateral to the QC are the epidermis/lateral root cap initials that, like the ground tissue initial, generate two tissues (Fig. 1) (Dolan *et al.*, 1993).

**Fig. 2.**
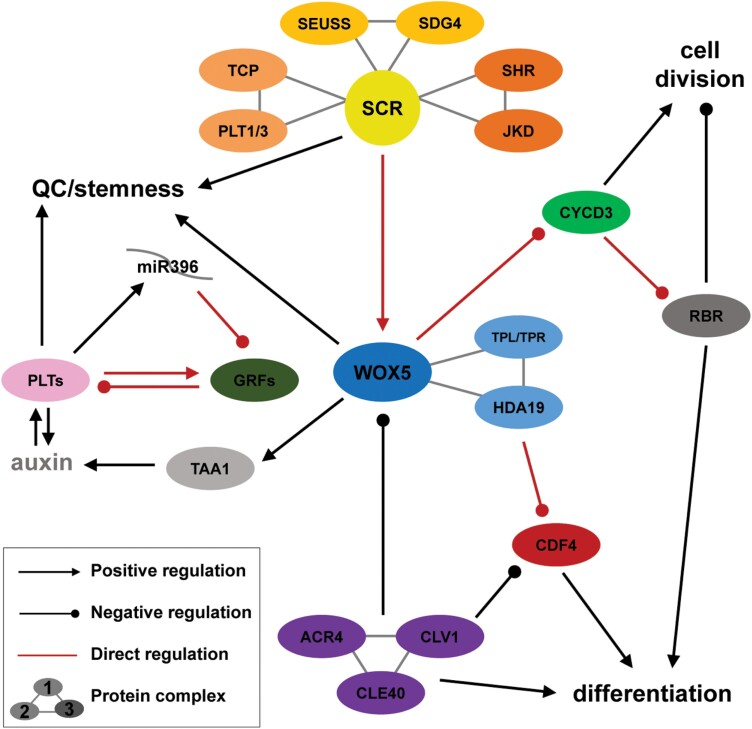
Overview of the pathways regulating the stem cell niche. At the top are three protein complexes for which SCR is fundamental. These complexes are implicated in the direct positive transcriptional regulation of *WOX5* in the QC. Note that data only support the assembly of SCR into individual complexes. Also, SCR can promote stemness and QC fate independently from WOX5. On the right is shown *CYCD3*, the expression of which is directly repressed by WOX5 in the QC and which participates in the phosphorylation of RBR, thereby inactivating it. Whereas CYCD3 promotes cell division, RBR—in its active form—represses cell division and promotes cell differentiation. On the left is shown *TAA1*, the expression of which is up-regulated by WOX5 and which in turn enhances auxin production within the QC. High levels of auxin increase PLT expression, which in turn promotes auxin production in a feed-forward loop. PLTs are required for QC specification and promote stemness of surrounding initials. In a feed-back loop, PLTs positively regulate expression of *GRF* and *miR396*, which directly post-transcriptionally repress *GRFs* in the SCN. Low levels of GRF can no longer directly repress *PLTs*, thereby assuring their high expression in the SCN. In the centre, WOX5 autonomously promotes QC fate and non-autonomously promotes stemness in the columella initials. WOX5 also forms a complex with TPL/TPR and HDA19 to repress the differentiation factor gene *CDF4* in the QC. Shown at the bottom, the ACR4–CLV1–CLE40 signalling module represses *WOX5* expression, whereas it promotes columella differentiation in a CDF4-independent manner. The red lines indicate direct transcriptional, post-transcriptional, or post-translational regulation based on experimental data.

Throughout this review, we dissect the complex puzzle underlying the specification and maintenance of the Arabidopsis root SCN into separate regulatory pieces. By exploring previous and recent data, we address the interaction between well-known factors such as PLETHORA, SCARECROW, and WOX5 in the establishment of a functional root organizing centre. Likewise, the relevance of intercellular communication within the SCN, the importance of quiescence, and the role of local auxin production are addressed. In addition, we explore the multiple complexes that have SCARECROW as a member, hypothesizing how these are formed and regulated within the cellular environment. Furthermore, we discuss in a comprehensive way how these networks connect and converge into a functional SCN (Table 1; Fig. 2).

**Table 1.** Main genes involved in SCN regulation

Gene	Description	Expression in post-embryonic root	SCN-related processes	References
*WOX5*	Homeobox TF	Strongly expressed in QC and weakly expressed in the proximal meristem	QC specification and maintenance; maintenance of columella initials	Sarkar *et al.* (2007), Forzani *et al.* (2014), Pi *et al.* (2015), Shimotohno *et al.* (2018), Berckmans *et al.* (2020), Zhai *et al.* (2020)
*CYCD3;3*	Cyclin	Lateral root cap, stele, distal columella, and epidermal initials	Columella formation and maintenance of columella initials	Forzani *et al.* (2014)
*RBR1*	Retinoblastoma protein family	Whole root tip	QC quiescence; columella differentiation; ground tissue formative divisions	Wildwater *et al.* (2005), Wachsman *et al.* (2011), Cruz-Ramírez *et al*. (2012),Cruz-Ramírez *et al*. (2013)
*SCR*	GRAS family TF	U-domain	QC specification and maintenance; ground tissue formative divisions	Sabatini (2003), Cui *et al.* (2007), Cruz-Ramírez *et al*. (2012), Hirano *et al.* (2017), Long *et al.* (2017), Shimotohno *et al.* (2018), Zhai *et al.* (2020)
*SHR*	GRAS family TF	Expressed in the stele; protein moves to U-domain	QC specification and maintenance; ground tissue formative divisions	Helariutta *et al.* (2000), Nakajima *et al.* (2001), Cui *et al.* (2007), Welch *et al.* (2007), Long *et al.* (2015), Long *et al.* (2017)
*BIB*	BIRD C2H2-type zinc finger protein	QC, ground tissue initials, and mature cells	QC maintenance; ground tissue formative divisions	Long *et al.* (2015)
*JKD*	BIRD C2H2-type zinc finger protein	QC, ground tissue initials and mature cells	QC specification and maintenance; maintenance of columella initials; ground tissue formative divisions	Welch *et al.* (2007), Long *et al.* (2015), Hirano *et al.* (2017), Long *et al.* (2017)
*PLT’s*	AP2/ERF TF	Diverse gradient patterns; all peaking at the SCN	QC/SCN specification and maintenance	Aida *et al.* (2004), Galinha *et al.* (2007), Mähönen *et al*. (2014), Santuari *et al.* (2016)
*miR396*	MicroRNA	Combined miR396A and miR396B: QC, lateral root cap, columella initials, and mature cells	SCN maintenance	Rodriguez *et al.* (2015)
*CDF4*	Dof zinc finger protein	Columella initials and mature cells	Columella differentiation	Pi *et al.* (2015)
*ACR4*	Receptor kinase	Part of lateral root cap, columella initials, and mature cells	Columella differentiation	Stahl *et al.* (2009), Stahl *et al.* (2013), Berckmans *et al.* (2020)
*CLV1*	Receptor kinase	Part of lateral root cap, columella initials, and mature cells	Columella differentiation	Stahl *et al.* (2013), Berckmans *et al.* (2020)
*CLE40*	CLV3/ESR signal peptide family	Differentiating stele and columella	Columella differentiation	Stahl *et al.* (2009), Stahl *et al.* (2013), Berckmans *et al.* (2020)
*HDA19*	Histone deacetylase	Whole root tip; reduced expression in the columella	Maintenance of columella initials	Pi *et al.* (2015)
*SDG4*	Histone-lysine methyltransferase	SCN	QC specification and maintenance; maintenance of columella initials	Kumpf *et al.* (2014), Zhai *et al.* (2020)
*SEU*	LIM-domain-binding co-regulator	Whole root tip	QC specification and maintenance; maintenance of columella initials	Gong *et al.* (2016), Zhai *et al.* (2020)
*TCP20/TCP21*	TCP family TF	Combined TCP20 and TCP21: whole root tip	QC specification and maintenance; maintenance of columella initials	Shimotohno *et al.* (2018)
*TPL/TPR*	TOPLESS co-repressor	TOPLESS is expressed in the whole root tip; reduced expression in the columella	Maintenance of columella initials	Pi *et al.* (2015)
*TAA1*	Tryptophan aminotransferase	QC	Auxin biosynthesis; SCN maintenance	Stepanova *et al.* (2008), Brumos *et al.* (2018), Savina *et al.* (2020)

TF, transcription factor.

## WOX5 autonomous function

When we refer to the QC, the first gene that comes to mind is likely to be *WUSCHEL RELATED HOMEOBOX 5* (*WOX5*). Expression of *WOX5* begins at the globular stage of embryogenesis, in the hypophyseal cell. Upon division of the hypophyseal cell, *WOX* expression is restricted to the lens shaped cell that is the founder of the QC (Haecker *et al.*, 2004; Sarkar *et al.*, 2007). Post-embryonically, the promoter of this transcriptional factor is highly active in the QC and slightly active in the vascular initials. However, driving the expression of a green fluorescent protein (GFP)–WOX5 fusion protein under its endogenous promoter has shown that the WOX5 protein also localizes to the adjacent columella initial layer, indicating its mobile nature (Pi *et al.*, 2015). The loss of function mutant *wox5-1* displays accumulation of starch in the columella initials, indicating that these cells fail to maintain their undifferentiated state. On the other hand, plants ectopically expressing *WOX5* develop multiple undifferentiated columella cell layers (Sarkar *et al.*, 2007; Pi *et al.*, 2015). In addition, *wox5-1* also presents extra transverse divisions in the QC (Sarkar *et al.*, 2007; Forzani *et al.*, 2014). Importantly, there is only a clear effect on proximal stem cell differentiation when *wox5-1* mutant is combined with mutations in *SHORT-ROOT* (*SHR*), *SCARECROW* (*SCR*), or *PLETHORA1* (*PLT1*) and *PLT2* genes (Sarkar *et al.*, 2007). Therefore, these data indicate that the primary roles of WOX5 are the autonomous prevention of QC divisions and the non-autonomous maintenance of columella stem cell activity (Sarkar *et al.*, 2007; Forzani *et al.*, 2014; Pi *et al.*, 2015).

RETINOBLASTOMA-RELATED (RBR) is a master regulator of the cell cycle. In its hypophosphorylated and active form, it prevents cell cycle progression. When phosphorylated by cyclin–cyclin-dependent kinase (CYC–CDKs) complexes, RBR is inactivated allowing cell cycle advancement (Boniotti and Gutierrez, 2001). Concordantly, *CYCLIN B1;1* expression was observed frequently in QC cells of *RBR* RNA interference plants, indicating that QC cells divide more frequently than in the wild type (WT). These plants also developed additional columella stem cell layers. However, these did not originate by enhanced stem cell divisions, indicating a role for RBR in stem cell differentiation. This was corroborated by ectopic RBR expression that had the opposite effect and resulted in differentiation of columella stem cells (Wildwater *et al.*, 2005). Using a clonal deletion system, it was shown that RBR confers its QC division and stem cell differentiation effects in a cell-autonomous manner (Wachsman *et al.*, 2011). Tissue specific *CYCLIN D* genes are candidates to modulate local RBR activity. Indeed, it has been shown that the ectopic expression of *CYCLIN D3;3* (*CYCD3;3*) under the *WOX5* promoter induces divisions in the QC and even leads to reduction in the expression of the QC identity markers *QC25* and *QC46* (Sabatini, 2003; Forzani *et al.*, 2014). Analysis of GFP reporter lines showed that *CYCD3;3* is expressed in the QC of *wox5-1* mutant but not in the QC of WT plants, indicating that the correct expression of *WOX5* is sufficient to exclude *CYCD3;3* expression from the QC. These results are further supported by chromatin immunoprecipitation (ChIP) experiments indicating that WOX5 binds to the *CYCD3;3* promoter (Forzani *et al.*, 2014). Together, these results demonstrate that at least part of the autonomous role of WOX5 in QC function is maintenance of quiescence through the regulation of *CYCD3;3*.

## How important is quiescence?

The abolition of QC quiescence leading to partial loss of QC identity either in the *wox5-1* mutant or upon ectopic *CYCD3;3* expression in the QC, suggests that the QC and its function in stem cell maintenance strictly depend on quiescence (Sarkar *et al.*, 2007; Forzani *et al.*, 2014). However, other mutants and conditions that induce ectopic divisions in the QC indicate otherwise. For example, the mutant *ethylene overproducer 1* (*eto1*) that overproduces ethylene, displays extra QC divisions but does not lose expression of the QC identity marker *QC25* (Ortega-Martínez *et al*., 2007). Similarly, four mutants insensitive to abscisic acid (*aba insensitive 1* (*abi1)*, *abi2*, *abi3*, and *abi5*) also exhibit enhanced QC divisions, but unlike *wox5-1* they do not lose the columella initials (Zhang *et al.*, 2010). Furthermore, despite the QC divisions observed upon RBR depletion from the QC, these roots still express *WOX5*, and in normal conditions, neither root growth nor root SCN function is affected. Nevertheless, differences are seen when plants are treated with a DNA-damaging agent. Upon loss of quiescence, the RBR-depleted QC is more sensitive to DNA damage (displayed by higher lethality), and over time, root growth is more affected compared with WT plants (Cruz-Ramírez *et al*., 2013). These observations indicate that quiescence is not essential for maintenance of a functional QC or root apical meristem, since a dividing QC can still function as an organizing centre. Thus, it appears that the main function of WOX5 is the non-autonomous maintenance of stem cells. On the other hand, the QC quiescence that is mediated autonomously by WOX5, through the direct regulation of *CYCD3* and indirect regulation of RBR, functions to protect the QC from stresses, exemplified by DNA damage induction experiments. In line with this, AP2/ERF subfamily X transcription factors ETHYLENE RESPONSE FACTOR 109 (ERF109) and ERF115 are induced by stress conditions, allowing the QC to divide in order to replenish affected stem cells (Heyman *et al.*, 2013; Kong *et al.*, 2018). ERF115 interacts with RBR thereby coupling at least part of its effect to the RBR–SCR module in regulating QC division (see below) (Zhou *et al.*, 2019). These observations expand the idea that the QC acts as a reservoir, ready to replenish damaged stem cells when required, thereby securing a sustained root meristem.

## QC communication is a two-way street

Investigation of the cell–cell communication in the stem cell niche using a modified version of the inducible callose deposition system (*icals3m*) (Vatén *et al*., 2011) strongly suggests that bilateral symplastic communication between the QC and the columella initials is essential for both QC and distal stem cell maintenance (Liu *et al.*, 2017). Blocking QC symplastic traffic by *WOX5* promoter-driven *icals3m* led to starch accumulation in QC and columella stem cells. In addition, QC cell division and misexpression of identity markers Q0608 (columella), J2341 (columella stem cells), and QC25 (QC) was observed. Interestingly, *proWOX5::erGFP* expression was not strongly reduced upon plasmodesmatal closure, suggesting that QC function is partially retained. These observations indicate that correct SCN function requires mutual and bilateral exchange of signals between QC and surrounding cells, rather than unilateral signalling from QC to stem cells. Nevertheless, the disorganized SCN that resulted from symplastic traffic disruption barely had an impact on meristem cell number or root growth, further supporting the idea that the SCN acts as a stem cell reservoir to support long-lasting root growth (Liu *et al.*, 2017). These results provide an elegant molecular elaboration on early ablation experiments indicating the essential role of positional cues for fate determination (van den Berg *et al.*, 1995, 1997).

## WOX5 as a non-autonomous factor

To test the requirement of WOX5 mobility for columella stem cell maintenance, Pi and collaborators generated a non-mobile version of WOX5 fused to three copies of yellow fluorescent protein (YFP). Expressed from its own promoter, WOX5–3xYFP was unable to prevent the phenotype of starch accumulation in the columella initials when introduced in the *wox5-1* mutant background. Despite this stem cell differentiation phenotype, expression of the QC identity marker *QC184* was restored in the *wox5-1* mutant indicating the WOX5–3xYFP fusion protein was functional. These results indicate that WOX5 mobility is required for columella stem cell maintenance but not for QC fate (Pi *et al.*, 2015). To elucidate the function of WOX5 in stem cell maintenance, transcriptomics, ChIP, and protein–protein interaction experiments were performed. These revealed that WOX5 binds to the promoter region of *CYCLING DOF FACTOR 4* (*CDF4*), where it forms a complex with TOPLESS/TOPLESS-RELATED (TPL/TPR) and HISTONE DEACETYLASE 19 (HDA19) proteins, leading to *CDF4* transcriptional repression. CDF4 induces stem cell differentiation, as shown by transgenic lines that accumulate starch in columella stem cells upon ectopic expression of *CDF4* either in the QC or in the columella initials. Interestingly, *CDF4* exhibits a gradient expression pattern, peaking at the mature columella cells, displaying low levels in the columella initials and no expression in the QC. Based on these observations, an elegant model was proposed in which WOX5 and CDF4 form opposite gradients. In this model, CDF4 promotes the differentiation of columella cells whereas WOX5 autonomously represses CDF4 expression in the QC and non-autonomously in the columella initials, maintaining their stem cell status (Pi *et al.*, 2015).

Recently, results that contradict the requirement of WOX5 mobility for columella stem cell maintenance were presented by Berckmans *et al.* (2020). In a similar *wox5-1* complementation experiment with an immobilized WOX5, but now fused to two GFPs, the QC and columella stem cell defects were restored to WT levels (Berckmans *et al.*, 2020). In their hands, also WOX5 fused to three GFPs was able to rescue the mutant phenotype, albeit partially. It was hypothesized that fusing WOX5 to the larger 3xGFP tag impaired its function, contradicting the previous observation of the fusion to 3xYFP that appeared to be generally functional in rescuing QC fate characteristics (Pi *et al.*, 2015; Berckmans *et al.*, 2020). Thus, it appears that a non-mobile, QC-localized WOX5 is sufficient for columella stem cell maintenance. In light of these data, stem cell maintenance warrants an independent factor from the QC to maintain stemness.

Columella differentiation is actively promoted by the CLAVATA3/ESR-RELATED40 (CLE40) peptide, which is expressed in the differentiating stele and columella cells, through its interaction with ARABIDOPSIS CRINKLY4 (ACR4) and CLAVATA1 (CLV1) receptor kinases. Single mutants *cle40*, *acr4*, and *clv1* exhibit an extra layer of columella initials, and the *acr4* mutant is insensitive to exogenous CLE40 treatment. Both receptors were shown to co-localize to the plasmodesmata, and form homo- and heterodimers (Stahl *et al.*, 2009, 2013). Based on these observations, a ‘gating model’ was hypothesized: CLE40 secreted from differentiated columella cells binds and activates ACR4–CLV1 complexes located at the plasmodesmata, thereby blocking the traffic of ‘stemness’ factors between the QC/columella initials and mature columella cells (Stahl *et al.*, 2013). However, recent experiments with exogenous CLE40 treatment did not appear to change the diffusion rate of fluorescent proteins between QC, columella initials, and mature cells. Also, WOX5–GFP mobility was not affected in *cle40*, *acr4*, or *clv1* single mutants (Berckmans *et al.*, 2020). These results indicate the CLE40–CLV1–ACR4 signalling module promotes columella differentiation independent of symplastic protein traffic control.

Upon CLE40 treatment, QC and columella initials accumulate starch; *WOX5* and QC markers (*QC184* and *ALG42*) are repressed in the QC and their expression relocated into vascular cells (Stahl *et al.*, 2013; Berckmans *et al.*, 2020). Interestingly, this shift of *WOX5* expression towards the vasculature as a prelude to QC re-specification is not accompanied by an apical rearrangement of the *CDF4* expression domain into the differentiating cells. This indicates that columella cells can also undergo differentiation in the absence of CDF4 (Pi *et al.*, 2015; Berckmans *et al.*, 2020).

The multiple undifferentiated columella cell layers that form upon induced overexpression of *WOX5* fused to a glucocorticoid receptor (WOX5–GR) (Sarkar *et al.*, 2007) could be caused by de-differentiation of mature columella cells, by ectopic cell division, or by both. Treating WOX5–GR-induced overexpression plants with differentiation-inducing CLE40 peptide indicated that only the first mature columella layer may undergo de-differentiation (Berckmans *et al.*, 2020). However, reproduction of the WOX5–GR-inducible overexpression experiments with a focus on cell division dynamics suggested that the observed multiple stem cell phenotype is exclusively caused by proliferation of columella stem cells (Savina *et al.*, 2020), which are subsequently maintained in their undifferentiated state.

In summary, contradictory data on the WOX5 mobility requirement for non-autonomous distal meristem maintenance highlighted the requirement of a downstream stemness factor from the QC.

## Local auxin is key

For a long time it has been known that auxin is implicated in the control of patterning of the root SCN (Sabatini *et al.*, 1999; Blilou *et al.*, 2005). Exogenous auxin application (or ectopic auxin production) represses *WOX5* and induces columella differentiation. This process depends on the transcriptional repressor AUXIN RESISTANT3 (IAA17/AXR3) and on two AUXIN RESPONSE FACTORS, ARF10 and ARF16, which were suggested to confine *WOX5* expression to the QC (Ding and Friml, 2010). To address the importance of local auxin biosynthesis and transport for correct SCN function, a double mutant displaying impaired auxin biosynthesis in shoots and roots (*wei8;tar2*) (Stepanova *et al.*, 2008) was used in grafting experiments (Brumos *et al.*, 2018). By fusing WT shoots to *wei8;tar2* mutant roots, it was demonstrated that the shoot-produced auxin that is transported to the root is unable to restore the root SCN function. The characteristic WT auxin distribution gradient, with an auxin maximum in the QC and lower auxin levels in mature columella cells, was not re-established in these chimeric plants, and consequently the root meristem was terminated. The same result was observed when full *wei8;tar2* mutant plant shoots were locally treated with exogenous auxin. Even though the shoot treatment induced formation of adventitious and lateral roots, the root apical meristem was not maintained (Brumos *et al.*, 2018). Conversely, the auxin gradient and SCN function of *wei8;tar2* mutant roots was restored by local exogenous auxin application or by expression of auxin biosynthesis genes in the root. However, complementation occurred under the condition that the PIN-FORMED auxin efflux carriers were not disturbed. This shows that in roots locally supplied by an auxin source, the auxin transporters have the capacity to create the auxin maximum in the QC. When polar auxin transport was blocked, only QC-specific (*WOX5* promoter driven) auxin biosynthesis was able to maintain the root meristem, confirming the importance of an auxin peak in the QC for SCN function (Brumos *et al.*, 2018). These data agree with previous findings that upon ectopic callose-induced closure of QC plasmodesmata, the auxin gradient is lost and starch accumulation is observed in QC and columella initials. Apparently, disrupting QC symplastic traffic impairs local auxin biosynthesis, also evidenced by down-regulation of auxin biosynthetic genes. Again, exogenous auxin treatment or QC-specific promoter driven expression of a bacterial auxin biosynthesis gene rescued the QC and columella stem cell defects (Liu *et al.*, 2017).

Upon induction of ectopic *WOX5-GR* expression, prior to any morphological change, the *TRYPTOPHAN AMINOTRANSFERASE OF ARABIDOPSIS1* (*TAA1*) gene that is involved in auxin biosynthesis is up-regulated and has its expression domain expanded into the columella (Savina *et al.*, 2020). A similar pattern was observed for the auxin polar transporters PIN1 and PIN4, suggesting that *WOX5* ectopic expression impacts both auxin biosynthesis and transport. Mathematical modelling supported the hypothesis that WOX5-induced TAA1-mediated auxin biosynthesis is sufficient to explain the phenotypes observed in the columella. Absence of QC quiescence and the presence of one or two stem cells were predicted for a 50% reduction in TAA1-dependent synthesis in a WT background (Savina *et al.*, 2020). Accordingly, *wox5-1* might be an *in vivo* representation of this predicted phenotype, since it displays QC divisions, and indeed it was shown to have reduced *TAA1* marker expression. Finally, the model predicted that a 50% reduction of TAA1-dependent auxin biosynthesis in a *WOX5* overexpression line restores the columella phenotype back to WT. This was confirmed by partial restoration using a competitive inhibitor of TAA1-dependent auxin synthesis. Altogether these results reveal locally produced auxin to be a candidate for a non-autonomous signal originating in the QC, downstream of WOX5, to regulate distal meristem functioning (Savina *et al.*, 2020).

## SHORT-ROOT, SCARECROW, and JACKDAW: a ground tissue detour

SCR and SHR are two transcription factors from the GRAS family that are well known for their role in regulating the asymmetric division of the ground tissue stem cell daughter that generates cortex and endodermis. Single null mutants for either *SCR* or *SHR* (*scr*, *shr*) fail to perform the asymmetric cell division of the ground tissue initial daughter, ending up with a single layer of ground tissue, which has cortical characteristics in *shr* and mixed cortical–endodermal features in *scr* (Benfey *et al.*, 1993; Scheres *et al.*, 1995; Di Laurenzio *et al.*, 1996; Helariutta *et al.*, 2000). Previous work has shown that *SHR* is expressed in the stele, and its encoded protein moves one layer outward into the so-called U-domain (composed by QC, ground tissue initials, and endodermis), where it physically interacts with SCR. By forming a complex, SCR was reported to promote SHR nuclear localization, which leads to direct up-regulation of *SCR* and prevents SHR from moving to outer layers, thus restricting it to the U-domain (Helariutta *et al.*, 2000; Nakajima *et al.*, 2001; Cui *et al.*, 2007). Several zinc-finger proteins of the C2H2 type, belonging to the so-called BIRD family, such as JACKDAW (JKD), NUTCRACKER (NUC), BALD-IBIS (BIB), and MAGPIE (MGP), which are expressed in the ground tissue, also play important roles in this process. For example, the *jkd* mutant displays ectopic cortical periclinal divisions, a phenotype intensified when *JKD*’s homolog *BIB* is knocked down in the *jkd* background (*jkd;bib-i*) (Welch *et al.*, 2007; Long *et al.*, 2015). In *jkd;bib-i*, *SCR* expression and SHR nuclear retention are compromised, which correlates to spreading of SHR to multiple layers outside its transcription domain. In line with that, it was shown that JKD and BIB can promote SHR nuclear localization, a process that is reinforced by SCR (Long *et al.*, 2015).

The process regulating the formative division to generate cortex and endodermis is a puzzle that also involves RBR, CYCLIN D6;1 (CYCD6;1), CYCLIN-DEPENDENT KINASE B1 (CDKB1), and CDKA1, and additionally auxin (Sozzani *et al.*, 2010; Cruz-Ramírez *et al*., 2012; Weimer *et al.*, 2012). *CYCD6;1* expression is directly and specifically up-regulated by the SHR–SCR complex in cells that undergo asymmetric cell division such as the ground tissue initial daughter. Consistently, null *cycd6;1* mutants exhibit delayed cortex–endodermis formative divisions, while ectopic transactivation of *CYCD6;1* in ground tissue produces an extra endodermal layer (Sozzani *et al.*, 2010). RBR forms a ternary complex with SHR–SCR repressing some of their targets (i.e. *MGP*, *NUC*, and *CYCD6;1*). Consistently, *scr* complementation with SCR^AxCxA^ (which has reduced binding to RBR) leads to expansion of the *CYCD6;1* expression domain and the formation of an extra QC and ground tissue layer. In turn, CYCD6;1 interacts with CDKB1;1 and CDKB1;2 to phosphorylate and inactivate RBR, preventing RBR from co-repressing SCR targets, thereby forming a bistable circuit. SHR–SCR complex activity together with auxin-induced CYCD6;1 accumulation forms the basis of restricting the ground tissue formative division to the stem cell daughter (Cruz-Ramírez *et al*., 2012). Another study suggests that CYCD6;1 primarily acts through CDKA;1 to promote the formative divisions, in a dose-dependent manner. Accordingly, intermediate levels of CDKA activity were shown to be sufficient to promote symmetric cell division, whereas high levels of CDKA activity are required to completely inactivate RBR inducing formative divisions. This study also suggests that CDKBs would have a minor role in the formative division process, just backing up CDKA;1 function (Weimer *et al.*, 2012). Dual luciferase reporter assays in Arabidopsis protoplasts have shown that the up-regulation of *CYCD6;1* by SCR–SHR is counteracted by JKD and BIB, which matches the observations that the *CYCD6;1* domain is expanded in *jkd;bib-i* roots. These results indicate that JKD and BIB restrict *CYCD6;1* expression to ground tissue initials and daughters, preventing its expression in already differentiated endodermal and cortical cells (Long *et al.*, 2015).

The concept of proteins being assembled into different complexes in order to promote specific processes is further supported and extended by Förster resonance energy transfer measured by fluorescence lifetime microscopy (FRET-FLIM) data. FRET-FLIM experiments show that SCR and SHR association is stronger in the ground tissue initial/daughter cells that undergo asymmetric cell division, compared with QC or endodermis. On the other hand, JKD–SCR association is stronger in endodermal cells compared with QC or ground tissue initial/daughter. In addition, a split-luciferase assay in HeLa cells demonstrated that addition of JKD competes with the SCR–SHR interaction, making it weaker, whereas SHR addition makes the SCR–JKD association stronger. Importantly, co-immunoprecipitation (CoIP) experiments in tobacco leaves show that SCR, SHR, and JKD can form a ternary complex, indicating that the FRET results may represent either the formation of distinct heterodimers or the assembly of a ternary complex with closer interaction between two of the partners (Long *et al.*, 2017). Indeed, the crystal structure for a SHR–SCR–JKD complex suggests that, at least in the formation of this ternary complex, JKD only binds to SHR and not directly to SCR (Hirano *et al.*, 2017). These and other results reinforce the idea that differential interactions between SHR, SCR, and JKD triggers different processes: whereas strong SHR–SCR interaction is associated with ground tissue asymmetric cell division, stronger SCR–JKD interaction is associated with endodermis specification.

## Wasn’t this about SCN? Yes, QC function also requires SHR, SCR, and JKD

In a no less complex process, SHR, SCR, and JKD are also pivotal for QC specification and maintenance. Also, the heat shock transcription factor gene *SCHIZORIZA* (*SCZ*) was shown to genetically interact with *SCR* and *SHR* for SCN and ground tissue patterning, although the underlying mechanism remains unclear (Mylona *et al.*, 2002; Pernas *et al.*, 2010; ten Hove *et al.*, 2010). The single mutants *shr*, *scr*, *scz*, and *jkd* display defective QC specification and maintenance, exemplified by a disorganized stem cell niche, accumulation of starch in the columella initials, and failure to correctly express QC identity markers such as QC25 and QC46. All four mutants have shorter meristems and roots, a phenotype that is less prominent in *jkd*, while *shr* and *scr* are unable to maintain root meristematic activity (Benfey *et al.*, 1993; Scheres *et al.*, 1995; Helariutta *et al.*, 2000; Sabatini, 2003; Pernas *et al.*, 2010; ten Hove *et al.*, 2010; Welch *et al.*, 2007). Whereas *SCR* expression is reduced in the *shr* mutant, transactivation of *SCR* in the QC of *shr* or *scr* only rescued the root stem cell niche defect of *scr*, indicating that both genes are required for correct QC specification and maintenance (Sabatini, 2003). It was also observed that from early heart stage onward, *SCR* is no longer expressed in *jkd* mutants, while *JKD* expression is only reduced in *shr* and *scr* mutants after embryogenesis. Taken together, SHR and JKD are required for *SCR* expression during and after embryogenesis, whereas SHR and SCR are only required for elevated *JKD* expression in post-embryonic roots. Furthermore, analysis of double mutants showed that *jkd;scr* displays a stronger phenotype compared with the *scr* single mutant, but *jkd;shr* has only slightly shorter roots than *shr*, suggesting that JKD exerts its function in root stem cell niche mostly through SHR (Welch *et al.*, 2007).

The same FRET-FLIM experiments that showed stronger association of SHR–SCR and SCR–JKD in ground tissue initial/daughter and endodermal cells, respectively, have also shown that SHR and JKD associate more strongly in QC cells. Interestingly, whereas *WOX5* expression is mostly absent in *shr*, *scr*, and *jkd* mutants, ectopic *WOX5* promoter-driven *JKD* expression leads to expansion of the *WOX5* expression domain towards vasculature. Given that the *WOX5* promoter is by default weakly active in vascular initials, a feedforward loop was suggested to amplify *WOX5* promoter activity in the vasculature. This effect was observed also in *scr* and *shr* backgrounds, indicating that ectopically expressed JKD alone can induce *WOX5* expression. The enlargement of the *WOX5* domain coincided with expansion of the QC identity markers *QC25*, *QC46*, and *QC184*, indicating that JKD is able to promote QC fate. Concordantly, dual-luciferase reporter experiments in tobacco leaves demonstrated that among all possible combinations, SHR–JKD was the one to more effectively enhance *WOX5* promoter activity. Additionally, split-luciferase in HeLa cells indicated that SCR strengthens the association between SHR and JKD. Put together, these results suggest that, whether by forming a SHR–JKD heterodimer or by assembly of a ternary complex with SCR in a supporting role, an enhanced interaction between SHR and JKD is associated with QC specification and maintenance via *WOX5* regulation (Long *et al.*, 2017).

## PLETHORA proteins maintain the QC in a redundant fashion

Another set of transcriptional factors necessary for QC specification and maintenance are the PLT proteins. Four PLTs (PLT1–4) have partially overlapping expression domains in the form of a gradient, peaking in the root stem cell niche. In addition, PLTs are also partially redundant in function, acting in a dose-dependent manner. Whereas single mutants do not display obvious phenotypes, *plt1;plt2* double mutants display a strongly reduced root meristem that undergoes terminal differentiation, and *plt1;plt2;plt3* triple mutants completely fail to develop a primary root (Aida *et al.*, 2004; Galinha *et al.*, 2007). Consistently, *plt1;plt2* double mutants fail to correctly express the QC identity markers *QC25*, *QC46*, and *QC184*, and accumulate starch granules in the columella initials (Aida *et al.*, 2004). Interestingly, despite the resemblance of *plt1:plt2* mutants to *scr* and *shr* regarding the stem cell niche defects, *SHR* and *SCR* expression is not affected in *plt1;plt2* mutants and vice versa. Concordantly, *scr;plt1;plt2* and *shr;plt1;plt2* triple mutants display earlier root meristem differentiation when compared with *shr/scr* single- or *plt1;plt2* double-mutants (Aida *et al.*, 2004). Collectively, these results suggest that PLTs and SHR/SCR act in parallel pathways converging to specify and maintain the QC and the root stem cell niche (Aida *et al.*, 2004; Galinha *et al.*, 2007; Santuari *et al.*, 2016). *PLTs* were shown to be induced by auxin and to directly or indirectly regulate the expression of many genes involved in patterning, growth, and differentiation, including genes involved in auxin biosynthesis, response, and transport (Aida *et al.*, 2004; Mähönen *et al*., 2014; Santuari *et al.*, 2016). Besides the auxin–PLT feedforward cascade, additional PLT-regulated genes feed back on *PLTs*, thereby creating additional feedforward loops (Santuari *et al.*, 2016). The GROWTH-REGULATING FACTOR–GRF-INTERACTING FACTOR (GRF–GIF) repressive module, consisting of interacting transcriptional repressors and co-regulators, is a candidate to control PLT levels through negative feedback (Rodriguez *et al.*, 2015; Santuari *et al.*, 2016; Ercoli *et al.*, 2018). PLTs promote *GRF* and *GIF* expression, whereas GIF co-regulators are implicated in the direct repression of *PLTs* (Santuari *et al.*, 2016; Ercoli *et al.*, 2018). *miR396* post-transcriptionally represses *GRFs*, whereas PLTs maintain high levels of *miR396* within the SCN. Therefore *miR396* excludes the GRFs from the SCN, restricting their activity to the transit-amplifying zone (Rodriguez *et al.*, 2015; Ercoli *et al.*, 2018). This interplay between PLT, GIF*–*GRF, and *miR396* collaborates to establish the PLT gradients, consisting of reduced levels in the transit-amplifying zone and maxima at the SCN.

## Parallel pathways converge in the QC

A recent study has described for the first time how PLT and SCR parallel pathways may converge into QC specification (Shimotohno *et al.*, 2018). By means of yeast-two-hybrid (Y2H) and bimolecular fluorescence complementation (BiFC) in protoplasts, PLT1, PLT3, and SCR were shown to interact with two TEOSINTE-BRANCHED/CYCLOIDEA/PCNA proteins (TCP20 and TCP21). To map the interaction domains, Y2H and BiFC experiments were subsequently performed using truncated versions of TCP20 and TCP21, showing that PLTs and SCR bind to different regions of the TCPs. Additionally, CoIP in protoplasts transiently expressing tagged PLT3, TCP20, and SCR combinations showed that PLT3 and SCR only interact in the presence of TCP20. These results suggest that SCR, PLTs, and TCPs form a complex *in planta*. To understand the individual and combined dose effect of these genes on root development, several double, triple, and quadruple mutants were obtained, varying the allelic contribution of these four genes. For example, a double mutant homozygous for the *scr* allele and heterozygous for *tcp20* (*scr;tcp20+/−*) displays an intermediary root length compared with a *scr* single mutant (longer root) and the double homozygous *scr;tcp20* mutant (shorter root). A quadruple homozygous *plt1;plt3;tcp20;scr3* mutant completely abolishes primary root growth (Shimotohno *et al.*, 2018). The results are consistent with these genes acting in a dose-dependent manner for root length.

As expected, these mutants also present morphological defects in the root stem cell niche. The quadruple homozygous mutant completely loses the stereotypical stem cell niche pattern, whereas triple homozygous (*plt1;tcp20;scr* and *plt3;tcp20;scr*) mutants exhibit extra QC divisions and accumulate starch in columella initials earlier than observed in the *scr* single mutant. Morphological changes are already observed early during embryogenesis: quadruple homozygous mutants exhibit unusual divisions in the hypophyseal cell at the dermatogen stage, corresponding to the time and position of their expression. Concordantly, the *WOX5* promoter is less active in a double *tcp;scr* compared with *scr* single mutant, and less expressed in quadruple heterozygous mutants compared with WT (Shimotohno *et al.*, 2018). These results again reveal a gene dose output response, consistent with protein complex formation.

ChIP-seq data reported the occurrence of PLT2 binding at the *WOX5* promoter (Santuari *et al.*, 2016), suggesting that PLT1 and PLT3 may be mediating the output of the complex through direct regulation of *WOX5* target expression. Supporting this hypothesis, it was demonstrated that PLT1 or PLT3 fused to GR, upon dexamethasone induction and in the presence of the protein synthesis inhibitor cycloheximide, were able to directly induce *WOX5* expression. Mutating the PLT binding sites in the *WOX5* promoter abolished its expression, indicating the relevance of the identified binding sites. Dual-luciferase assays in protoplasts showed that, although induced by PLT3 alone, *WOX5* promoter activity was significantly enhanced when PLT3, TCP20, and SCR were combined. In addition, compared with WT promoter induction by PLT3–TCP20–SCR, the induction of the promoter harbouring mutated PLT-binding motifs was markedly reduced. Altogether, these data strongly support the idea that PLT proteins together with SCR form a TCP-containing complex in order to specify the QC and establish the root stem cell niche (Shimotohno *et al.*, 2018).

Recently, Zhai and collaborators presented data supporting *WOX5* regulation by SCR in a PLT1/2-independent manner, thereby elaborating on earlier data that indicated that *WOX5* expression depends mainly on *SCR/SHR*, with *PLT1/PLT2* playing only a minor role in confining WOX5 expression to the QC (Sarkar *et al.*, 2007; Zhai *et al.*, 2020). Previous data showed reduced fluorescence of *SHR* and *SCR* promoter and protein fusions in a *seuss* (*seu*) mutant background (Gong *et al.*, 2016). The nuclear localized SEU protein is a transcriptional co-regulator that is broadly expressed in embryos and in post-embryonic root meristem. Indeed, the *scr;seu* and *shr;seu* double mutants do not display enhanced phenotypes compared with *scr* and *shr* single mutants. However, a *seu;plt1;plt2* triple mutant presents shorter roots compared with *plt1;plt2*. These results suggest that SCR/SHR and SEU act in the same pathway in parallel to the PLT pathway (Gong *et al.*, 2016).

*WOX5* expression is both delayed and significantly reduced in a *seu* null mutant. Accordingly, *seu* displays phenotypes similar to the *wox5* mutant, harbouring ectopic divisions in the QC, starch accumulation in columella initials, and strongly reduced expression of the *QC184* identity marker. Also, *seu;wox5* double mutants did not present enhanced defects compared with *wox5*, indicating that both genes act in the same pathway (Zhai *et al.*, 2020). Interestingly, Y2H showed that SEU interacts with SCR but not SHR and the SCR–SEU interaction was confirmed by CoIP in Arabidopsis roots. ChIP–qPCR experiments showed enrichment of SCR and SEU binding at ~1100 bp upstream of the *WOX5* transcription start site, with dramatically reduced binding for SEU in a *scr* mutant background. Also, dual-luciferase assays in tobacco leaves showed that *WOX5* promoter activity is ~15% up-regulated by the SCR–SEU combination compared with SCR alone. These results indicate that SCR recruits and physically interacts with SEU to jointly bind to the *WOX5* promoter (Zhai *et al.*, 2020).

As a third player in this complex, the histone methyltransferase protein SET DOMAIN GROUP 4 (SDG4) was shown to interact with SEU by Y2H and split-luciferase in tobacco leaves. The null mutant *sdg4* (originally *ashr3-1*) displays defects resembling those observed in *seu* (Kumpf *et al.*, 2014). Concordantly, the SDG4–GFP fusion protein was observed during embryo development from dermatogen stage onwards in the nucleus of the hypophysis, whereas post-embryonically it was broadly expressed in the root meristem. SDG4 and SCR bind to different regions of SEU as shown by Y2H, while CoIP in tobacco leaves and Arabidopsis roots demonstrated that SCR and SDG4 can associate to SEU at the same time, and that SEU is required for SCR–SDG4 interaction. ChIP–qPCR experiments for SDG4–GFP showed enrichment of the same region in the *WOX5* promoter as is observed to be bound by GFP–SCR and SEU–GFP. In addition, *sdg4;seu;wox5* and *sdg4;seu;scr* triple mutants do not enhance QC defects observed in either single *wox5* or *scr* mutant, respectively. Furthermore, WOX5–GFP overexpression is able to rescue the QC defects in a *sdg4;seu;scr* triple mutant. Finally, both s*dg4* and *seu* mutants display reduced levels of H3K4me3 marks (associated with transcriptional activation) at the *WOX5* promoter. Altogether, these results support a SCR–SEU–SDG4 complex that triggers H3K4 trimethylation of the *WOX5* promoter, in order to promote its expression and consequently QC specification (Zhai *et al.*, 2020).

## Wrap up: the attractive SCARECROW

The research discussed above indicates the existence of at least three ternary complexes regulating QC specification and *WOX5* expression. All of these include the SCR transcription factor interacting with two partners (PLT/TCP, SEU/SDG4 or SHR/JKD) (Long *et al.*, 2017; Shimotohno *et al.*, 2018; Zhai *et al.*, 2020). The corresponding genes start to be expressed early during embryogenesis, around the time of or prior to *WOX5* accumulation at the globular stage (Helariutta *et al.*, 2000; Welch *et al.*, 2007; Shimotohno *et al.*, 2018; Zhai *et al.*, 2020). Realizing that all these proteins display overlapping expression domains in the hypophysis/QC, it is intriguing to think how those complexes behave in this same cellular environment. Do all complexes co-exist or are there factors regulating the conditional assembly of one complex at the expense of the others? Considering that SCR is a key factor for all complexes, at least some competition could be expected. The reported protein–protein interaction experiments so far only addressed which SCR domain is used for the interaction with SHR, but not for the interaction with JKD, TCP20/21, and SEU (Hirano *et al.*, 2017; Long *et al.*, 2017; Shimotohno *et al.*, 2018; Zhai *et al.*, 2020). The evidence presented in literature only addresses and supports the assembly of individual complexes. However, we can speculate that if SCR uses different domains for its interactions, one unique SCR might associate to more than one complex at the same time. In line with this, it was previously demonstrated that SCR binds to RBR through a domain that is dispensable for its interaction with SHR (Cruz-Ramírez *et al*., 2012). Therefore, we can hypothesize on at least two possible scenarios. Considering that SCR appears to be crucial for recruitment and assembly of a SEU complex at ~1100 bp as well as for the TCP complex ~300 bp upstream of the *WOX5* transcription start site (Shimotohno *et al.*, 2018; Zhai *et al.*, 2020), we can envision that SCR physically links both PLT–TCP and SDG4–SEU sub-complexes, and maybe even the SHR–JKD sub-complex, and mediates their interaction in the formation of a single multi-protein complex (Shimotohno *et al.*, 2018; Zhai *et al.*, 2020). In this way, the SDG4–SEU sub-complex would promote H3K4me3 methylation, making the *WOX5* promoter region more accessible to other factors, including the PLT–TCP sub-complex, to effectively induce *WOX5* expression. On the other hand, in the case of SCR only being assembled into separate complexes, those different complexes may perform specific roles during different subsequent phases of QC specification and *WOX5* expression, thereby recycling SCR between the different complexes. For example, the SDG4–SEU–SCR complex could primarily establish chromatin activation marks at *WOX5* promoter, and this would relax the chromatin allowing subsequent complexes such as JKD–SHR–SCR and PLT–TCP–SCR to access this region in order to promote and maintain *WOX5* expression during and after embryogenesis.

The question is whether SCR itself can physically accommodate binding to all these proteins or whether there is a common additional scaffold protein(s) to accommodate all sub-complexes, or perhaps there is a hybrid version of these proposed mechanisms. In that respect it is also interesting to examine the role of the Mediator complex for which the subunit MED31 has been observed to compete with SHR for binding to SCR, in order to regulate downstream *CYCD6;1* transcription in the ground tissue (Zhang *et al.*, 2018). The large Mediator complex couples the enhancer-bound transcription factors to RNA polymerase II-dependent gene transcription, which includes epigenetic control of transcription (Buendía-Monreal and Gillmor, 2016). One way of testing these hypotheses is to determine whether SCR, while being part of one complex, can bind to proteins of the other complexes. A first candidate would be SHR, since the interaction between SCR and SHR was reported to be stronger than binding of SCR to JKD, RBR, or MED31 (Cruz-Ramírez *et al*., 2012; Long *et al.*, 2017; Zhang *et al.*, 2018). In addition, since SHR can enhance the association between JKD and SCR, SHR may act in stabilizing the interaction between SCR and other complexes (Long *et al.*, 2017). Whereas these hypotheses are still purely speculative, future experiments will tell whether SCR can act as a core protein being assembled into multiple complexes simultaneously, or if SCR is recycled between complexes to exert its function in the QC and SCN regulation. Since the first ablation studies that showed the importance of organizer signalling for stem cell maintenance, detailed studies have provided insights into genes and networks acting in the SCN. Nevertheless, more is still to be discovered on the mechanisms acting in the short-range control of SCN function and stem cell differentiation.

## References

[CIT0001] Aida M , Beis D, Heidstra R, Willemsen V, Blilou I, Galinha C, Nussaume L, Noh YS, Amasino R, Scheres B. 2004. The *PLETHORA* genes mediate patterning of the *Arabidopsis* root stem cell niche. Cell 119, 109–120. 15454085 10.1016/j.cell.2004.09.018

[CIT0002] Benfey PN , Linstead PJ, Roberts K, Schiefelbein JW, Hauser MT, Aeschbacher RA. 1993. Root development in *Arabidopsis*: four mutants with dramatically altered root morphogenesis. Development 119, 57–70. 8275864 10.1242/dev.119.Supplement.57

[CIT0003] Berckmans B , Kirschner G, Gerlitz N, Stadler R, Simon R. 2020. CLE40 signaling regulates root stem cell fate. Plant Physiology 182, 1776–1792. 31806736 10.1104/pp.19.00914 PMC7140941

[CIT0004] Blilou I , Xu J, Wildwater M, Willemsen V, Paponov I, Friml J, Heidstra R, Aida M, Palme K, Scheres B. 2005. The PIN auxin efflux facilitator network controls growth and patterning in *Arabidopsis* roots. Nature 433, 39–44. 15635403 10.1038/nature03184

[CIT0005] Boniotti MB , Gutierrez C. 2001. A cell-cycle-regulated kinase activity phosphorylates plant retinoblastoma protein and contains, in Arabidopsis, a CDKA/cyclin D complex. The Plant Journal 28, 341–350. 11722776 10.1046/j.1365-313x.2001.01160.x

[CIT0006] Brumos J , Robles LM, Yun J, Vu TC, Jackson S, Alonso JM, Stepanova AN. 2018. Local auxin biosynthesis is a key regulator of plant development. Developmental Cell 47, 306–318.e5. 30415657 10.1016/j.devcel.2018.09.022

[CIT0007] Buendía-Monreal M , Gillmor CS. 2016. Mediator: A key regulator of plant development. Developmental Biology 419, 7–18. 27287881 10.1016/j.ydbio.2016.06.009

[CIT0008] Cruz-Ramírez A , Díaz-Triviño S, Blilou I, et al. 2012. A bistable circuit involving SCARECROW-RETINOBLASTOMA integrates cues to inform asymmetric stem cell division. Cell 150, 1002–1015. 22921914 10.1016/j.cell.2012.07.017 PMC3500399

[CIT0009] Cruz-Ramírez A , Díaz-Triviño S, Wachsman G, et al. 2013. A SCARECROW-RETINOBLASTOMA protein network controls protective quiescence in the Arabidopsis root stem cell organizer. PLoS Biology 11, e1001724. 24302889 10.1371/journal.pbio.1001724 PMC3841101

[CIT0010] Cui H , Levesque MP, Vernoux T, Jung JW, Paquette AJ, Gallagher KL, Wang JY, Blilou I, Scheres B, Benfey PN. 2007. An evolutionarily conserved mechanism delimiting SHR movement defines a single layer of endodermis in plants. Science 316, 421–425. 17446396 10.1126/science.1139531

[CIT0011] Di Laurenzio L , Wysocka-Diller J, Malamy JE, Pysh L, Helariutta Y, Freshour G, Hahn MG, Feldmann KA, Benfey PN. 1996. The SCARECROW gene regulates an asymmetric cell division that is essential for generating the radial organization of the *Arabidopsis* root. Cell 86, 423–433. 8756724 10.1016/s0092-8674(00)80115-4

[CIT0012] Ding Z , Friml J. 2010. Auxin regulates distal stem cell differentiation in *Arabidopsis* roots. Proceedings of the National Academy of Sciences, USA 107, 12046–12051. 10.1073/pnas.1000672107 PMC2900669 20543136

[CIT0013] Dolan L , Janmaat K, Willemsen V, Linstead P, Poethig S, Roberts K, Scheres B. 1993. Cellular organisation of the *Arabidopsis thaliana* root. Development 119, 71–84. 8275865 10.1242/dev.119.1.71

[CIT0014] Ercoli MF , Ferela A, Debernardi JM, Perrone AP, Rodriguez RE, Palatnik JF. 2018. GIF transcriptional coregulators control root meristem homeostasis. The Plant Cell 30, 347–359. 29352064 10.1105/tpc.17.00856 PMC5868699

[CIT0015] Forzani C , Aichinger E, Sornay E, Willemsen V, Laux T, Dewitte W, Murray JA. 2014. WOX5 suppresses CYCLIN D activity to establish quiescence at the center of the root stem cell niche. Current Biology 24, 1939–1944. 25127220 10.1016/j.cub.2014.07.019 PMC4148176

[CIT0016] Galinha C , Hofhuis H, Luijten M, Willemsen V, Blilou I, Heidstra R, Scheres B. 2007. PLETHORA proteins as dose-dependent master regulators of *Arabidopsis* root development. Nature 449, 1053–1057. 17960244 10.1038/nature06206

[CIT0017] Gong X , Flores-Vergara MA, Hong JH, Chu H, Lim J, Franks RG, Liu Z, Xu J. 2016. SEUSS integrates gibberellin signaling with transcriptional inputs from the SHR-SCR-SCL3 module to regulate middle cortex formation in the Arabidopsis root. Plant Physiology 170, 1675–1683. 26818732 10.1104/pp.15.01501 PMC4775121

[CIT0018] Haecker A , Gross-Hardt R, Geiges B, Sarkar A, Breuninger H, Herrmann M, Laux T. 2004. Expression dynamics of *WOX* genes mark cell fate decisions during early embryonic patterning in *Arabidopsis thaliana*. Development 131, 657–668. 14711878 10.1242/dev.00963

[CIT0019] Helariutta Y , Fukaki H, Wysocka-Diller J, Nakajima K, Jung J, Sena G, Hauser MT, Benfey PN. 2000. The *SHORT-ROOT* gene controls radial patterning of the *Arabidopsis* root through radial signaling. Cell 101, 555–567. 10850497 10.1016/s0092-8674(00)80865-x

[CIT0020] Heyman J , Cools T, Vandenbussche F, et al. 2013. ERF115 controls root quiescent center cell division and stem cell replenishment. Science 342, 860–863. 24158907 10.1126/science.1240667

[CIT0021] Hirano Y , Nakagawa M, Suyama T, Murase K, Shirakawa M, Takayama S, Sun TP, Hakoshima T. 2017. Structure of the SHR-SCR heterodimer bound to the BIRD/IDD transcriptional factor JKD. Nature Plants 3, 17010. 28211915 10.1038/nplants.2017.10 PMC5639936

[CIT0022] Kong X , Tian H, Yu Q, et al. 2018. PHB3 maintains root stem cell niche identity through ROS-responsive AP2/ERF transcription factors in *Arabidopsis*. Cell Reports 22, 1350–1363. 29386120 10.1016/j.celrep.2017.12.105

[CIT0023] Kumpf R , Thorstensen T, Rahman MA, et al. 2014. The ASH1-RELATED3 SET-domain protein controls cell division competence of the meristem and the quiescent center of the Arabidopsis primary root. Plant Physiology 166, 632–643. 25034019 10.1104/pp.114.244798 PMC4213094

[CIT0024] Liu Y , Xu M, Liang N, Zheng Y, Yu Q, Wu S. 2017. Symplastic communication spatially directs local auxin biosynthesis to maintain root stem cell niche in *Arabidopsis*. Proceedings of the National Academy of Sciences, USA 114, 4005–4010. 10.1073/pnas.1616387114 PMC5393224 28348232

[CIT0025] Long Y , Smet W, Cruz-Ramírez A, et al. 2015. *Arabidopsis* BIRD zinc finger proteins jointly stabilize tissue boundaries by confining the cell fate regulator SHORT-ROOT and contributing to fate specification. The Plant Cell 27, 1185–1199. 25829440 10.1105/tpc.114.132407 PMC4558684

[CIT0026] Long Y , Stahl Y, Weidtkamp-Peters S, et al. 2017. In vivo FRET-FLIM reveals cell-type-specific protein interactions in *Arabidopsis* roots. Nature 548, 97–102. 28746306 10.1038/nature23317

[CIT0027] Mähönen AP , Ten Tusscher K, Siligato R, Smetana O, Díaz-Triviño S, Salojärvi J, Wachsman G, Prasad K, Heidstra R, Scheres B. 2014. PLETHORA gradient formation mechanism separates auxin responses. Nature 515, 125–129. 25156253 10.1038/nature13663 PMC4326657

[CIT0028] Mylona P , Linstead P, Martienssen R, Dolan L. 2002. *SCHIZORIZA* controls an asymmetric cell division and restricts epidermal identity in the *Arabidopsis* root. Development 129, 4327–4334. 12183384 10.1242/dev.129.18.4327

[CIT0029] Nakajima K , Sena G, Nawy T, Benfey PN. 2001. Intercellular movement of the putative transcription factor SHR in root patterning. Nature 413, 307–311. 11565032 10.1038/35095061

[CIT0030] Ortega-Martínez O , Pernas M, Carol RJ, Dolan L. 2007. Ethylene modulates stem cell division in the *Arabidopsis thaliana* root. Science 317, 507–510. 17656722 10.1126/science.1143409

[CIT0031] Pernas M , Ryan E, Dolan L. 2010. SCHIZORIZA controls tissue system complexity in plants. Current Biology 20, 818–823. 20417101 10.1016/j.cub.2010.02.062

[CIT0032] Pi L , Aichinger E, van der Graaff E, Llavata-Peris CI, Weijers D, Hennig L, Groot E, Laux T. 2015. Organizer-derived WOX5 signal maintains root columella stem cells through chromatin-mediated repression of *CDF4* expression. Developmental Cell 33, 576–588. 26028217 10.1016/j.devcel.2015.04.024

[CIT0033] Rodriguez RE , Ercoli MF, Debernardi JM, Breakfield NW, Mecchia MA, Sabatini M, Cools T, De Veylder L, Benfey PN, Palatnik JF. 2015. MicroRNA miR396 regulates the switch between stem cells and transit-amplifying cells in *Arabidopsis* roots. The Plant Cell 27, 3354–3366. 26645252 10.1105/tpc.15.00452 PMC4707450

[CIT0034] Sabatini S , Beis D, Wolkenfelt H, et al. 1999. An auxin-dependent distal organizer of pattern and polarity in the *Arabidopsis* root. Cell 99, 463–472. 10589675 10.1016/s0092-8674(00)81535-4

[CIT0035] Sabatini S , Heidstra R, Wildwater M, Scheres B. 2003. SCARECROW is involved in positioning the stem cell niche in the *Arabidopsis* root meristem. Genes & Development 17, 354–358. 12569126 10.1101/gad.252503 PMC195985

[CIT0036] Santuari L , Sanchez-Perez GF, Luijten M, et al. 2016. The PLETHORA gene regulatory network guides growth and cell differentiation in Arabidopsis roots. The Plant Cell 28, 2937–2951. 27920338 10.1105/tpc.16.00656 PMC5240741

[CIT0037] Sarkar AK , Luijten M, Miyashima S, Lenhard M, Hashimoto T, Nakajima K, Scheres B, Heidstra R, Laux T. 2007. Conserved factors regulate signalling in *Arabidopsis thaliana* shoot and root stem cell organizers. Nature 446, 811–814. 17429400 10.1038/nature05703

[CIT0038] Savina MS , Pasternak T, Omelyanchuk NA, Novikova DD, Palme K, Mironova VV, Lavrekha VV. 2020. Cell dynamics in WOX5-overexpressing root tips: the impact of local auxin biosynthesis. Frontiers in Plant Science 11, 560169. 33193486 10.3389/fpls.2020.560169 PMC7642516

[CIT0039] Scheres B , Laurenzio LD, Willemsen V, Hauser MT, Janmaat K, Weisbeek P, Benfey PN. 1995. Mutations affecting the radial organisation of the *Arabidopsis* root display specific defects throughout the embryonic axis. Development 121, 53–62.

[CIT0040] Shimotohno A , Heidstra R, Blilou I, Scheres B. 2018. Root stem cell niche organizer specification by molecular convergence of PLETHORA and SCARECROW transcription factor modules. Genes & Development 32, 1085–1100. 30018102 10.1101/gad.314096.118 PMC6075145

[CIT0041] Sozzani R , Cui H, Moreno-Risueno MA, Busch W, Van Norman JM, Vernoux T, Brady SM, Dewitte W, Murray JA, Benfey PN. 2010. Spatiotemporal regulation of cell-cycle genes by SHORTROOT links patterning and growth. Nature 466, 128–132. 20596025 10.1038/nature09143 PMC2967763

[CIT0042] Stahl Y , Grabowski S, Bleckmann A, et al. 2013. Moderation of Arabidopsis root stemness by CLAVATA1 and ARABIDOPSIS CRINKLY4 receptor kinase complexes. Current Biology 23, 362–371. 23394827 10.1016/j.cub.2013.01.045

[CIT0043] Stahl Y , Wink RH, Ingram GC, Simon R. 2009. A signaling module controlling the stem cell niche in *Arabidopsis* root meristems. Current Biology 19, 909–914. 19398337 10.1016/j.cub.2009.03.060

[CIT0044] Stepanova AN , Robertson-Hoyt J, Yun J, Benavente LM, Xie DY, Dolezal K, Schlereth A, Jürgens G, Alonso JM. 2008. TAA1-mediated auxin biosynthesis is essential for hormone crosstalk and plant development. Cell 133, 177–191. 18394997 10.1016/j.cell.2008.01.047

[CIT0045] ten Hove CA , Willemsen V, de Vries WJ, van Dijken A, Scheres B, Heidstra R. 2010. *SCHIZORIZA* encodes a nuclear factor regulating asymmetry of stem cell divisions in the *Arabidopsis* root. Current Biology 20, 452–457. 20171102 10.1016/j.cub.2010.01.018

[CIT0046] van den Berg C , Willemsen V, Hage W, Weisbeek P, Scheres B. 1995. Cell fate in the *Arabidopsis* root meristem determined by directional signalling. Nature 378, 62–65. 7477287 10.1038/378062a0

[CIT0047] van den Berg C , Willemsen V, Hendriks G, Weisbeek P, Scheres B. 1997. Short-range control of cell differentiation in the *Arabidopsis* root meristem. Nature 390, 287–289. 9384380 10.1038/36856

[CIT0048] Vatén A , Dettmer J, Wu S, et al. 2011. Callose biosynthesis regulates symplastic trafficking during root development. Developmental Cell 21, 1144–1155. 22172675 10.1016/j.devcel.2011.10.006

[CIT0049] Wachsman G , Heidstra R, Scheres B. 2011. Distinct cell-autonomous functions of *RETINOBLASTOMA-RELATED* in *Arabidopsis* stem cells revealed by the Brother of Brainbow clonal analysis system. The Plant Cell 23, 2581–2591. 21742994 10.1105/tpc.111.086199 PMC3226226

[CIT0050] Weimer AK , Nowack MK, Bouyer D, Zhao X, Harashima H, Naseer S, De Winter F, Dissmeyer N, Geldner N, Schnittger A. 2012. Retinoblastoma related1 regulates asymmetric cell divisions in *Arabidopsis*. The Plant Cell 24, 4083–4095. 23104828 10.1105/tpc.112.104620 PMC3517237

[CIT0051] Welch D , Hassan H, Blilou I, Immink R, Heidstra R, Scheres B. 2007. Arabidopsis JACKDAW and MAGPIE zinc finger proteins delimit asymmetric cell division and stabilize tissue boundaries by restricting SHORT-ROOT action. Genes & Development 21, 2196–2204. 17785527 10.1101/gad.440307 PMC1950858

[CIT0052] Wildwater M , Campilho A, Perez-Perez JM, Heidstra R, Blilou I, Korthout H, Chatterjee J, Mariconti L, Gruissem W, Scheres B. 2005. The *RETINOBLASTOMA-RELATED* gene regulates stem cell maintenance in *Arabidopsis* roots. Cell 123, 1337–1349. 16377572 10.1016/j.cell.2005.09.042

[CIT0053] Zhai H , Zhang X, You Y, Lin L, Zhou W, Li C. 2020. SEUSS integrates transcriptional and epigenetic control of root stem cell organizer specification. The EMBO Journal 39, e105047. 32926464 10.15252/embj.2020105047 PMC7560201

[CIT0054] Zhang H , Han W, De Smet I, Talboys P, Loya R, Hassan A, Rong H, Jürgens G, Paul Knox J, Wang MH. 2010. ABA promotes quiescence of the quiescent centre and suppresses stem cell differentiation in the Arabidopsis primary root meristem. The Plant Journal 64, 764–774. 21105924 10.1111/j.1365-313X.2010.04367.x

[CIT0055] Zhang X , Zhou W, Chen Q, Fang M, Zheng S, Scheres B, Li C. 2018. Mediator subunit MED31 is required for radial patterning of *Arabidopsis* roots. Proceedings of the National Academy of Sciences, USA 115, E5624–E5633. 10.1073/pnas.1800592115 PMC6004453 29844159

[CIT0056] Zhou W , Lozano-Torres JL, Blilou I, Zhang X, Zhai Q, Smant G, Li C, Scheres B. 2019. A jasmonate signaling network activates root stem cells and promotes regeneration. Cell 177, 942–956.e14. 30955889 10.1016/j.cell.2019.03.006

